# Identification, Antioxidant Capacity, and Matrix Metallopeptidase 9 (MMP-9) In Silico Inhibition of Haloarchaeal Carotenoids from *Natronococcus* sp. and *Halorubrum tebenquichense*

**DOI:** 10.3390/microorganisms11092344

**Published:** 2023-09-19

**Authors:** Mariana Delgado-Garcia, Osvaldo Gómez-Secundino, Jorge A. Rodríguez, Juan Carlos Mateos-Díaz, Marcelo Muller-Santos, Cristobal N. Aguilar, Rosa Maria Camacho-Ruiz

**Affiliations:** 1 Bioengineering Department, Instituto Tecnológico de Estudios Superiores de Monterrey, Campus Guadalajara, Zapopan 45201, Jalisco, Mexico; marianadelgadog@tec.mx; 2 Industrial Biotechnology, Centro de Investigación y Asistencia en Tecnología y Diseño del Estado de Jalisco, Zapopan 44270, Jalisco, Mexico; osgomez_al@ciatej.edu.mx (O.G.-S.); jrodriguez@ciatej.mx (J.A.R.); jcmateos@ciatej.mx (J.C.M.-D.); 3 Biochemistry and Molecular Biology Department, Universidade Federal do Paraná, Curitiba 81530-900, Brazil; marcelomuller@ufpr.br; 4 Food Research Department, Facultad de Ciencias Químicas, Universidad Autónoma de Coahuila, Saltillo 25280, Coahuila, Mexico; cristobal.aguilar@uadec.edu.mx

**Keywords:** UPLC-ESI-MS/MS, haloarchaea, MMP-9, antioxidant, bacterioruberin, docking

## Abstract

Natural pigments from haloarchaea are of great interest; bacterioruberin is the major pigment, it shows higher antioxidant power when compared with β-carotene. However, characterization of bacterioruberin and its isomers along with its antioxidant and the matrix metallopeptidase 9 (MMP-9) inhibition activities in extracts from *Natronoccoccus* sp. TC6 and *Halorubrum tebenquichense* SU10 was not previously described, being the aim of this work. The carotenoids profile was performed by UV-Vis spectrophotometry, thin-layer chromatography, nuclear magnetic resonance spectroscopy, and high-resolution mass spectrometry (UPLC-ESI-MS/MS). Antioxidant capacity was determined for DPPH, ABTS, and FRAP. In addition, MMP-9 inhibition was studied using docking simulations. The carotenoid profile of studied strains was composed of bacterioruberin, some derivatives like mono, bis, and tris anhydrobacterioruberin, and also some bacterioruberin cis isomers. The carotenoid pools showed antioxidant capacity for DPPH > ABTS > FRAP; *Natronococcus* sp. TC6 carotenoid pool was better for ABTS and DPPH, while *Halorubrum tebenquichense* SU10 carotenoid pool was better for FRAP. Additionally, docking and molecular dynamics suggest that bacterioruberin inhibits MMP-9 through hydrophobic interactions near the catalytic site. Bacterioruberin shows the higher binding energy of −8.3 (kcal/mol). The carotenoids profile of both strains was elucidated, their antioxidant activity and singular participation of each carotenoid on MMP-9 in silico inhibition were evaluated.

## 1. Introduction

Microbial pigments have been used in different areas like pharmaceutic, biomedic, cosmetic, or food, principally for their chemical and biological properties. Particularly, Haloarchaea (*Halobacteriaceae* and *Haloferacaceae* families), extreme microorganisms that grow in hypersaline environments, up to 4.5 M of NaCl, can produce and accumulate red pigments in their cell membrane [1,2]. In haloarchaea, bacterioruberin is the principal pigment, and its isomers and other derivatives possess 50 carbon atoms (C_50_) with a longer system (13) of conjugated double bonds and four hydroxyl groups. The accumulation of these in the lipid membrane is due to oxygen tension, and high light intensity and salinity as principal environmental inductors, allowing stabilization of the cell membrane under osmotic stress acting as a permeable barrier of oxygen and other molecules [3,4,5,6]. In the case of bacteria, bacterioruberin regulates membrane fluidity, particularly for *Arthrobacter* species [7]. Other carotenoids (C_50_) produced by haloarchaea are bacterioruberin precursors; the most synthetized are 2-isopentenyl-3,4-dehydrorhodopin (IDR), Bis-anydrobacterioruberin (BABR), and monoanhydrobacterioruberin (MABR) as well as other carotenoids (C_40_) although in low concentration, but principally β-carotene, lycopene, phytoene, lycopersene, and their isomers, these last being very important precursors for carotenogenesis [5,8,9]. Additionally, in haloarchaea, such as *Halobacterium salinarum*, bacterioruberin *cis* and *trans* isomers have been found [10]. These groups of haloarchaea carotenoids are part of the hidroxycarotenoids group, and until now, only 30 have been reported, the majority found in haloarchaea according to the carotenoid database (http://carotenoiddb.jp/, accessed on 16 September 2022). Haloarchaeal pigments have been identified and characterized using UV-Vis spectroscopy, but this method is very limited. Liquid chromatography coupled with mass spectrometers (LC-MS) accompanied by NMR are required for correct structure characterization, commonly HPLC-APCI-MS/MS is used for identification of bacterioruberin and its isomers [11,12,13].

The haloarchaeal pigments are an innovative biotechnological product. Various studies have found potential applications in biomedicine, antimicrobial activity, antioxidant activity, cancer therapy (inhibitory effect on MMP-9), antiviral, and others [3,14,15,16,17,18]. Bacterioruberin is the most abundant haloarchaeal pigment, and it showed the highest antioxidant power when compared with classic commercial carotenoids like β-carotene. Actually, the number of studies on haloarchaeal carotenoids is few compared with other microbial pigments like β-carotene or astaxanthin; the major efforts are focused on the characterization, principally on the controversy about the nature of the pathways for carotenogenesis in haloarchaea [9]. Some studies about bacterioruberin biological activities include their activity on cancer cell lines like HER-2 breast cancer cells, Hep-G2 hepatocyte carcinoma cells, among others [19]. The photoprotective activity of bacterioruberin on DNA damage from UV and gamma irradiation has been reported [20]. However, no biological activity is reported for bacterioruberin isomers [20]. Archaeal pigments have been studied far less than other microbial pigments in the context of pharmaceutical applications. Furthermore, no clinical trials have been reported [21]. Additionally, there is limited information available about the synthesis, production, and composition of carotenoids in most archaea. There are fewer studies regarding in silico analysis of their anticancer potential and the specific characterization or identification of the pool pigments, their interaction behavior, and the possible beneficial effects on human health. Fortunately, the anticancer potential could be approached by molecular docking; a tool that describes the interaction of molecules with the binding sites of proteins. The inhibition of MMP-9 is a possible field of application for bacterioruberin because antioxidants are reported as MMP-9 inhibitors [22].

For this reason, the objective of this study was to determine the composition of carotenoids present in lesser studied haloarchaea, such as *Natronococcus* sp. TC6 and *Halorubrum tebenquichense*. Additionally, we aimed to demonstrate the biotechnological applications of this pool of pigments as antioxidants and explore their in silico inhibition of MMP-9.

## 2. Materials and Methods

### 2.1. Strains

*Natronococcus* sp. TC6 and *Halorubrum tebenquichense* SU10 were used as the source of carotenoids. *Natronococcus* sp. TC6 was kindly donated by Prof. Jacques Baratti of the Biocatalysis and Fine Chemistry laboratory (UMR, CNRS 6111), Marseille, France; the strain was isolated from El Golea Sebkha, a salt lake on Algerian Sahara. *Halorubrum tebenquichense* SU10 belongs to the microorganisms collection of CIATEJ (Centro de Investigación y Asistencia en Tecnologia y Diseño del Estado de Jalisco, A.C., Guadalajara, Mexico), the strain was isolated from salt crystals collected on Uyuni saltern of Bolivia. The strains were grown in ATCC2185 ^®^ culture media (g/L: NaCl (250), MgSO_4_·7H_2_O (20), Na_2_C_6_H_5_O_7_ (3), KCl (2), yeast extract (3), tryptone (5)). The strains were cultivated at 37 °C, 250 rpm, pH 7.5 for 48 h. Finally, the culture was centrifuged at 13,000 rpm, 20 min at 4 °C to obtain the cellular pellet and was conserved in congelation at −20 °C. The reagents and solvents used for the analysis were from Sigma Aldrich ^®^ (St. Louis, MO, USA).

### 2.2. Carotenoid Pigments Extraction

Frozen cellular pellet was resuspended with methanol and acetone (7:3) and shaken for 4 h. Every 30 min, the pigment extract was collected and methanol and acetone (7:3, *v*/*v*) were added until the pellet was visually discolored. The carotenoid pigment was filtered, and rotary evaporated. Then, the pigment was dissolved in acetone and hexane (1:1, *v*/*v*) for salt elimination and a second filtration was performed. Finally, the carotenoid extract was dried with liquid nitrogen and conserved at −20 °C in darkness.

### 2.3. C50 Carotenoid Profile Analysis

#### 2.3.1. UV-Vis Spectroscopy

Pigment extract was dissolved in acetone and verified at 400–600 nm in an UV-Vis spectrophotometer, BioTek Eon^™^ (Winooski, VT, USA). The approximate content of total carotenoids was determined by measuring the optical density at 495 nm.

#### 2.3.2. Thin-Layer Chromatography

Pigment extract was analyzed by thin-layer chromatography (TLC) on silica gel plates using the following mixture of solvents as mobile phase: acetone:petroleum ether (20:80, *v*/*v*), methanol:chloroform (7:93, *v*/*v*) for bacterioruberin and monoanhydrobacterioruberin, and petroleum ether:diethyl ether (99:1 *v*/*v*) for β-carotene and lycopene. β-carotene (Sigma Aldrich^®^, St. Louis, MO, USA) was used as standard at a concentration of 1 mg/mL. The carotenoid content was analyzed based on retention factor (Rf) values.

### 2.4. Characterization of Carotenoids Produced by Natronococcus sp. TC6 and Halorubrum tebenquichense SU10

#### 2.4.1. Nuclear Magnetic Resonance (^1^H-NMR)

The dry pigment extract was dissolved with MeOH (2 mL), and the sample was analyzed in the Center of Nuclear Magnetic Resonance of Universidade Federal do Paraná, Brazil, on a Bruker DRX Avance III spectrometer operating at 9.5 T; using one-dimensional ^1^H spectroscopy. Data were acquired and processed with Topspin 3.2 software (Bruker GmbH, Silberstreifen, Germany).

#### 2.4.2. UPLC-ESI-MS/MS Analysis

The samples were analyzed using a Waters UPLC Acquity H Class (Milford, MA, USA, 2010), instrument equipped with a quaternary pump (UPQSM), autosampler injector (UPPDALTC), and eʎ PDA (UPPDALTC) linked to Water Xevo TQ-S micro mass spectrometer detector. Water MassLynx V4.1 software was used for data acquisition and processing. The mass spectrometer detector was operated in positive ESI mode, with capillary voltage at 4.0 kV, 50 V cone voltage, 350 °C desolvation temperature, 150 °C source temperature, and 1 V of collision energy. The pigment extract was separated. The dry pigment extract was dissolved in C_2_H_3_N/CH_3_OH/CH_2_O_2_ (70:30:0.5, *v*/*v*/*v*). The analysis was carried out using two solvent phases. Solvent phase A CH_2_O_2_:H_2_O (99.9:0.1, *v*/*v*) and phase B C_2_H_3_N:CH_3_OH (85:15, *v*/*v*), and concentration gradients of these phases were applied: Gradient 1 (A:25%–B:75% (1 min)), Gradient 2 (A:30%–B:70% (1.1 min)), Gradient 3 (A:50%–B:50% (5 min)), Gradient 4 (A:50%–B:50% (10 min)), Gradient 5 (A:95%–B:5% (12.5 min)), Gradient 6 (A:95%–B:5% (13.5 min)), Gradient 7 (A:25%–B:75% (13.75 min)), and Gradient 8 (A:25%–B:75% (18 min)). The mass spectra were recorded in full scan mode over the range *m*/*z* 400–800.

### 2.5. Bioprospection of Archaeal Carotenoids

The concentration of carotenoids in the pigment extract was quantified and adjusted to 50–1000 µM of carotenoids eluted with acetone. The antioxidant activity was performed using ABTS, DPPH as radicals, and ferric ion-reducing antioxidant power (FRAP), the results were reported as TROLOX equivalents (µM per mL of extract) using a standard curve of 15–1000 ppm.

#### 2.5.1. 1,1-Diphenyl-2-picrylhydrazyl (DPPH) Radical Scavenging Assay

Briefly, 1,1-Diphenyl-2-picrylhydrazyl (DPPH) radical was dissolved in 60 mM of ethanol and 193 µL of DPPH was mixed with 7 µL of carotenoid extracts (concentration of 50–1000 µM). The mixture (As) was homogenized using vortex and was incubated at 25 °C, 30 min in the dark. After, absorbance of the sample was measured at 517 nm. The solution of radical DPPH on ethanol was used as control (Ac).

#### 2.5.2. 2,2-Azino-bis-3-ethylbenzothiazoline-6-sulfonic acid (ABTS) Radical Scavenging Assay

A solution of 7 mM of ABTS (2,2-azino-bis-3-ethylbenzothiazoline-6-sulfonic acid) radical in deionized water and a solution of 2.45 mM potassium persulfate in water were prepared. Both solutions were mixed at 2:1 ratio (*v*/*v*) respectively, and incubated at 16 h in the dark for the formation of the radical ABTS by the oxidation of ABTS with potassium persulfate at room temperature. The absorbance of the solution was adjusted at 0.700 ± 0.02 at 734 nm. Then, 5 µL of the carotenoid extract (concentration of 50–1000 µM) was added to 195 µL of ABTS and absorbance was measured at 734 nm (As). Radical ABTS ethanol solution was used as control (Ac).

#### 2.5.3. Ferric Reduction Antioxidant Power Test (FRAP)

2,4,6-tripyridin-2-yl-1,3,5-triazine (TPTZ) was dissolved in 40 mM of HCl, and FeCl_3_ was dissolved in water and 0.3 M of acetate buffer, pH 3.6. The TPTZ and FeCl_3_ were mixed at 1:1 (*v*/*v*) and diluted in 0.3 M of acetate buffer, pH 3.6. The working solution (As) was 10 µL of the sample, and 290 µL of Fe-TPTZ incubated for 15 min at 37 °C. The absorbance was measured at 593 nm.

The scavenging activity was calculated using the following equation:

Antioxidant activity (%) = [1 − (As/Ac)] × 100.

#### 2.5.4. Trolox Equivalent Antioxidant Capacity

The scavenging activity was then calculated as Trolox equivalent antioxidant capacity. Trolox (6-Hydroxy-2,5,7,8-tetramethylchroman-2-carboxylic acid) was dissolved in ethanol and a standard curve was performed (15–1000 ppm).

Trolox equivalent antioxidant capacity was expressed as µM TROLOX eq/mL of extract, using the following equation:

Trolox equivalent antioxidant capacity = IC_50_ of Trolox (µM)/IC_50_ of sample in each radical (µM).

#### 2.5.5. Data Analysis

Statistical analysis was run using Statgraphics Centurion XVI version 16.1.18 (Statgraphics Centurion for Windows, StatPoint Technology, Inc., The Plains, VA, USA). Analysis of variance (ANOVA) was performed with a factorial analysis, strain and pigment concentration were the factors. Block means were compared using the Fisher’s least significance difference (LSD) multiple range test, calculated at 0.05 probability level of significance (*p* < 0.05).

#### 2.5.6. Docking Assays of Archaeal Carotenoid Fragments

**Figure 1:**
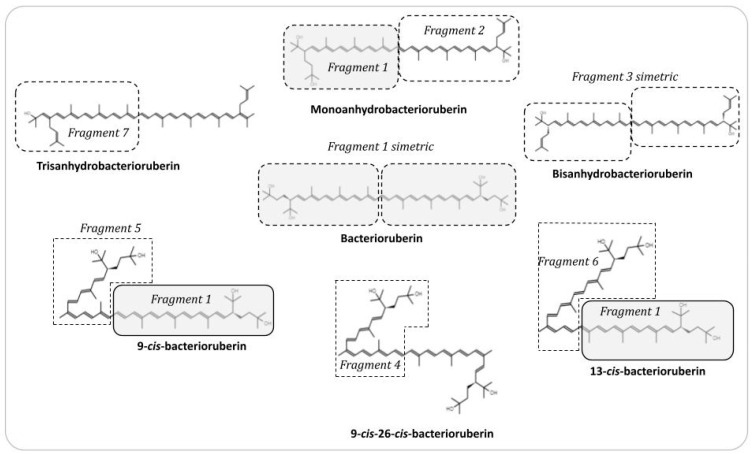
Carotenoid fragments structure found in *Natronococcus* sp. TC6 and *Halorubrum tebenquichense* SU10 and tested in docking assays. Each carotenoid fragment used in matrix metallopeptidase 9 (MMP-9) docking assays is in a box, the grey doted box indicates the fragment symmetric of bacterioruberin. The 2D chemical structures were made using JMSE editor web (https://biomodel.uah.es/en/DIY/JSME/draw.en.htm, accessed on 10 October 2022).

Docking simulation was carried out for each carotenoid fragment (Figure 1). The carotenoid fragments were constructed using JSME editor (https://biomodel.uah.es/en/DIY/JSME/draw.en.htm, accessed on 12 December 2022) and chemical structures are based on the work of Lizama et al. (2021) [16]. The geometries and partial charges (hydrogens were added according to pH 7.0) of each carotenoid fragment were optimized using Merck Force Field method (MMFF94) in Avogadro software v.1.2. The enzyme structure of matrix metallopeptidase 9 (MMP-9) (PDB: 1L6j) was downloaded from the Protein Data Bank RCSB PDB (https://www.rcsb.org/, accessed on 12 December 2022). Finally, docking experiments were performed using AutoDock Vina v.1.2.0 [23] and visualized using UCSF Chimera v.1.16. The grid map was calculated and centered on the putative catalytic site Glu402. The grid map volume was center: 35.5773, 49.6947, 47.7724, and size: 33.5661, 37.1846, and 23.6784 points, respectively. Additionally, the H-bonds and distances were calculated.

#### 2.5.7. Molecular Docking and Molecular Dynamics Simulation of MMP-9 and Bacterioruberin

The coordinates of MMP-9 were downloaded from Protein Data Bank (PDB entry 1L6J) and its structure was obtained from PubChem. A first-step molecular docking analysis was carried out with Vina v.1.2.5 and UCSF Chimera software v.1.17.3 to find the possible binding site of bacterioruberin. A box of size 30, 30, 30 with coordinates 42.40, 48.85, and 41.14 was generated for Binding Site 1; 38.43, 47.80, 56.58 was generated for Binding Site 2 and 25.67, 43.26, and 45.6 for “Allosteric Binding Site”.

Afterward, Classical molecular dynamics (MD) simulations were performed using the GROMACS 2023.2 package [24]. Protonation states were set, according to PROPKA3. All the input files were generated using the CHARMM-GUI web-based graphical interface. The CHARMM36m all-atom force field was used to generate topology [25,26]. Protein–ligand complex was solvated using the TIP3P water model in a rectangular box under periodic boundary conditions and 10 Å from the protein to the surface of the box: A, B, C as 94, 94, 94, and α, β, γ as 90, 90, 90, respectively. The system was neutralized by the ion placing method of Monte Carlo with counter ions of NaCl, reaching a final concentration of 150 mM. The equilibration step was carried out using the canonical ensemble NVT after the minimization step, and MD simulations were performed under NPT conditions at 303.15 K and 100 ns of trajectory [27]. GROMACS 2023.2, UCSF Chimera, Pymol, and VMD 1.9.1 were used to analyze the trajectories and visualize the simulations. The GROMACS 2023.2 software tool gmx_gyrate was used to determine the radius of gyration, and gmx_rmsf and gmx_rms were used to evaluate the RMSF and RMSD, respectively. The number of hydrogen bonds was calculated with gmx_hbond. All graphics were generated using Xmgrace software v.5.0.2 [28]. Pocket properties were calculated with Protein Plus Web Server and protein hydrophobic surface was calculated with UCSF Chimera.

## 3. Results

### 3.1. Identification of Carotenoids

#### 3.1.1. UV-Vis Spectrum and TLC Analysis

**Figure 2:**
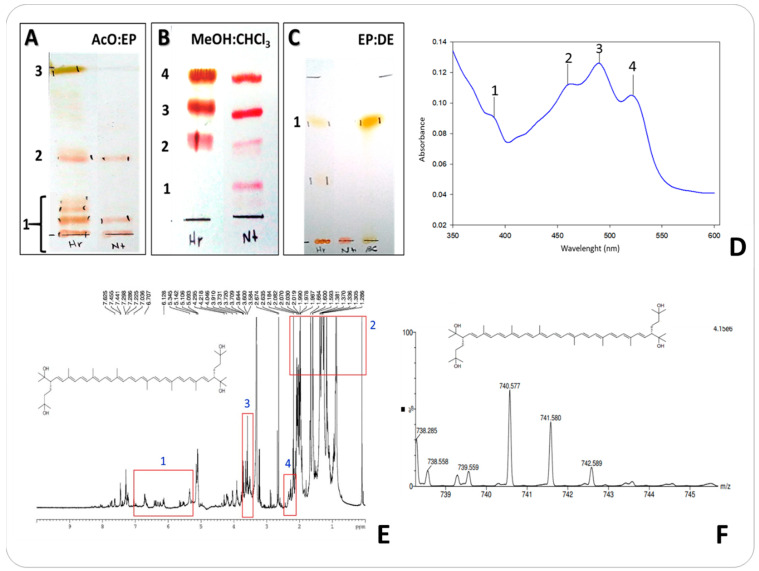
Carotenoid pigments in *Natronococcus* sp. TC6 and *Halorubrum tebenquichense* SU10. (**A**) TLC analysis of carotenoid pigments using acetone:petroleum ether (20:80, *v*/*v*) Hr: *Halorubrum tebenquichense,* Nt: *Natronococcus* sp. TC6, BC: β-carotene standard (1: bacterioruberin and derivatives, 2: non-identified, 3: Ɣ-carotene). (**B**) TLC analysis of carotenoid pigments using methanol:chloroform (7:93, *v*/*v*) (1: Bacterioruberin, 2–3: non-identified, 4: β-carotene). (**C**) TLC analysis of carotenoid pigments petroleum ether:diethyl ether (99:1 *v*/*v*) (1: β-carotene). (**D**) UV-Vis spectra of bacterioruberin profile (1 [384 nm], 2 [460 nm], 3 [490 nm], 4 [520 nm]). (**E**) ^1^H-NMR spectrum of bacterioruberin (1: hydrogens in the isoprene chain (5.5–6.8 ppm), 2: bacterioruberin typical signals (1–2 ppm), 3: hydroxyl groups (3.64 ppm), 4: double bound hydrogens (2.03–2.07 ppm). (**F**) UPLC-ESI-MS/MS spectra of bacterioruberin.

The pigment extracts from *Natronococcus* sp. TC6 and *Halorubrum tebenquichense* SU10 were analyzed at 300–600 nm, showing spectral peaks at 460, 490, and 520 nm (Figure 2D), the typical “three-finger”. The maximum absorption in 469, 494, and 526 nm corresponds to bacterioruberin.

The evidence of bacterioruberin (BAR) was also found in both strains analyzed by thin-layer chromatography, showing Rf of 0.08 (Figure 2A(1)) and 0.22 (Figure 2B, number 2) using acetone:petroleum ether (20:80, *v*/*v*) and methanol:chloroform (7:93, *v*/*v*) as mobile phase, respectively. Rf values not identified (0.09, 0.16, and 0.20) (Figure 2A, number 1) were found in *Halorburum tebenquichense* SU10; it is possible these correspond to bacterioruberin (BAR) derivates like monoanhydrobacterioruberin (MABR), bisanhydrobacterioruberin (BABR), and 2-isopentenyl-3,4 dihydrorhodopin (DIR). β-carotene was found in *Halorubrum tebenquichense* SU10 pigment extract (Figure 2A, number 4 and Figure 2C, number 1). On the other hand, pool pigment from *Natronococcus* sp. TC6 could be composed of possible bacterioruberin derivatives (Figure 2A,B).

#### 3.1.2. NMR and UPLC-ESI-MS/MS Analysis for Identification of Carotenoids Profile

Bacterioruberin was identified in both *Halorubrum tebenquichense* SU10 and *Natronococcus* sp. TC6 pigment extracts using NMR analysis (Figure 2E). Signals of the major compound in the pool pigments were revealed, the hydrogens in the isoprene chain of bacterioruberin correspond to signal 1 (5.5–6.8 ppm), signal 2 corresponds to CH_3_ groups (0.8–2 ppm), signal 3 is the hydroxyl group (3.64 ppm), and signal 4 is the double bound hydrogens (2.03–2.07 ppm) confirming the characteristic olefinic proton in aromatic and aliphatic regions in pool pigments. The presence of bacterioruberin (C_50_H_76_O_4_) was confirmed in both strains with the isotopic mass peaks corresponding to molecular weight of [M + H]^+^ *m*/*z* 740.5, 741.5, 742.5 (Table 1) from UPLC-ESI-MS/MS analysis (Figure 2F). Mass values and the most abundant MS/MS fragment ions obtained in positive mode for the different isolated carotenoids are shown in Table 1. Bacterioruberin derivates were identified, particularly, 2-isopentenyl-3,4 dihydrorhodopin IDR (C_45_H_64_O) at *m*/*z* 620.3 and monoanhydrobacterioruberin MABR (C_50_H_74_O_3_) at *m*/*z* 722.47 (Table 1), also identified by TLC and UV-Vis spectra analysis. Other pigments and intermediaries were identified: astaxanthin (C_40_H_52_O_4_) at *m*/*z* 598.39, β-carotene (C_40_H_56_) at *m*/*z* 537. 2, phytoene (C_40_H_64_) at *m*/*z* 543.50, and lycopersene (C_40_H_66_) at 545.96, some of these found in TLC analysis (Figure 2A–C).

### 3.2. Bioprospection of Pigment Extracts

#### 3.2.1. Antioxidant Activity

**Figure 3:**
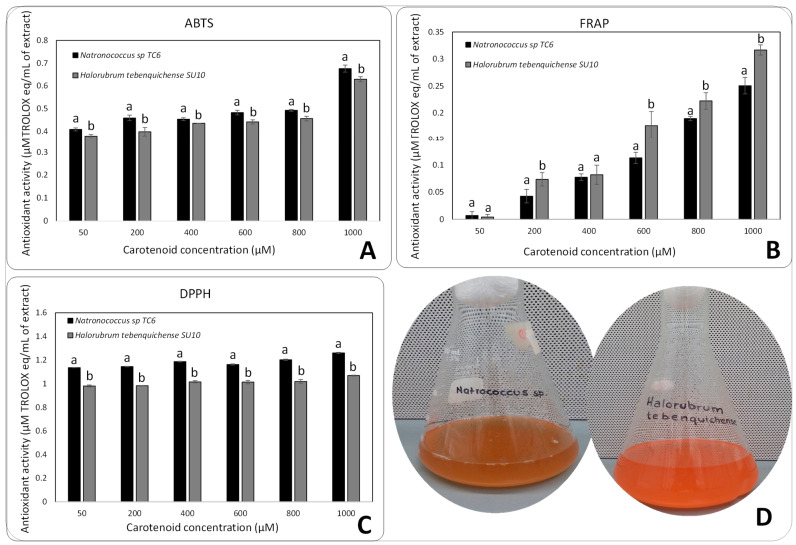
Antioxidant capacity of carotenoid extracts from *Natronococcus* sp. TC6 and *Halorubrum tebenquichense* SU10 tested for radical scavenging on DPPH, ABTS, and ferric ion-reducing antioxidant power (FRAP). (**A**) ABTS radical scavenging capacity of the carotenoid extracts. (**B**) Ferric ion-reducing antioxidant power (FRAP) of the carotenoid extracts. (**C**) DPPH radical scavenging capacity of the carotenoid extracts. (**D**) Pigmentation of strains *Halorubrum tebenquichense* SU10 and *Natronococcus* sp. TC6. Means with distinct letters represent statistical difference (*p* < 0.05) according to Fisher’s least significance difference (LSD) test related to the strain from which the pigment was extracted. Error bars represent Standard Deviation of three measures.

The antioxidant capacity of the pigment extracts of *Natronococcus* sp. and *Halorubrum tebenquichense* SU10 was tested by the radical scavenging of DPPH, ABTS, and FRAP (Figure 3). The pigment extracts showed considerable antioxidant activity, being able to scavenge all the radicals evaluated. Both *Halorubrum tebenquichense* SU10 and *Natronococcus* sp. TC6 pigment extracts showed scavenging activity on ABTS radical, 0.4 µM TROLOX equivalents/mL of extract using 50 µM of carotenoid extracts (Figure 3A). In the case of DPPH (Figure 3C), both carotenoid extracts showed an important ability to reduce the radical, the *Natronococcus* sp. TC6 carotenoid extract being slightly better (1.2 µM TROLOX equivalents/mL of extract using 50 µM of carotenoid extract). In the case of ferric ion-reducing antioxidant power FRAP (Figure 3B), *Halorubrum tebenquichense* SU10 showed a major antioxidant capacity of 0.31 µM/TROLOX equivalents/mL of extract using a 1000 µM solution of carotenoid extract. According to the statistical analysis, there are significant differences (*p* < 0.05) in the inhibition between the strains from which the pigments were extracted, *Natronococcus* sp. TC6 was better in ABTS and DPPH radicals and *Halorubrum tebenquichense* SU10 was better in FRAP. In in the case of FRAP, there are significant differences (*p* < 0.05) for extract pigment concentration (Figure 3B).

#### 3.2.2. Docking Assays of Archaeal Carotenoid Fragments

**Figure 4:**
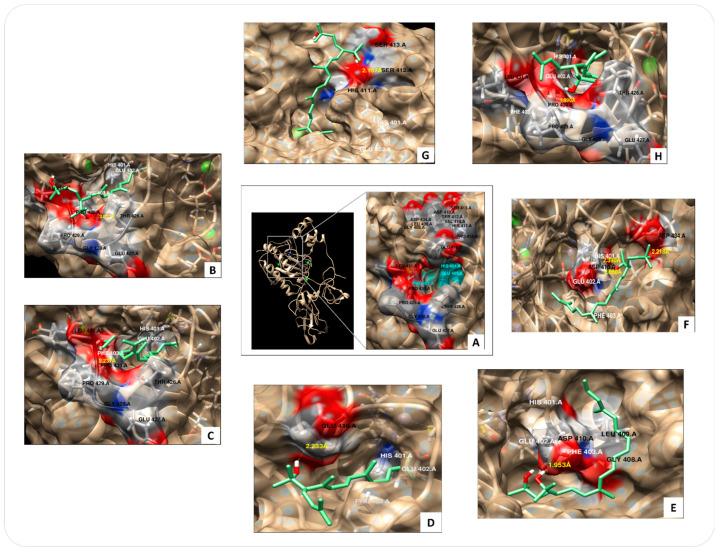
Docking assays of different archaeal carotenoid fragments over metalloproteinase 9 (MMP-9) (PDB: 1L6j). (**A**) MMP-9 catalytic pocket site. (**B**) Fragment 1 (bacterioruberin). (**C**) Fragment 2 (monoanhydrobacterioruberin). (**D**) Fragment 3 (bisanhydrobacterioruberin). (**E**) Fragment 4 (9-*cis*-26-*cis*-bacterioruberin). (**F**) Fragment 5 (9-*cis*-bacterioruberin). (**G**) Fragment 6 (13-*cis*-bacterioruberin). (**H**) Fragment 7 (trisanhydrobacterioruberin).

Docking assays were performed on MMP-9, which is an enzyme with 707 amino acid residues, consisting of five domains: signal sequence, prodomain, catalytic domain, fibronectin domain, and hemopexin domain. The catalytic domain contains two zinc and three calcium atoms and is characterized by conserved HEXXHXXGXXH binding motif. The carotenoid fragments evaluated over MMP-9 were selected based on the chemical skeleton of bacterioruberin in the plane symmetry and hydroxyl groups present (Figure 1). The carotenoid fragments (in green, Figure 4) have an interaction that favor possible inhibitory activity given by a bound consistently fitting into the enzyme pocket and near to the catalytic site, obtaining a free energy of about ΔG (kcal/mol) −6 to −8 values (Figure 4B–H), and showing a possible effect on the enzyme catalysis.

Table 2 shows the best docking binding energies (kcal/mol) of each fragment. Fragments of bacterioruberin, monoanhydrobacterioruberin, and bisanhydrobacterioruberin interact with different amino acids in the catalytic pocket.

A specific cavity is formed near to His401, Glu402, Phe403, and specifically interacts in the zone of Thr406, Glu427, Gly428, Pro429 and 430, and Gly431 (Figure 4B,C,H). Similarly, Fragment 3 of bisanhydrobacterioruberin interacts with Glu416, near to the catalytic pocket (Figure 4D). Contrary, 13-*cis*-bacterioruberin, 9-*cis*-26-bacterioruberin, and 9-*cis*-bacterioruberin form other binding site cavities, also near to the catalytic pocket and showed minor binding values (−6.8, −6.7 and −6.6 kcal/mol, respectively) (Figure 4E–G).

Fragments 1, 2, and 7 corresponding to bacterioruberin, monoahydrobacterioruberin, and trisanhydrobacterioruberin have the best binding energy values of −8.3, −8.2, and −8.3 kcal/mol, respectively (Figure 4B,C,H and Table 2). On the other hand, the 9-*cis*-26-bacterioruberin fragment has the lowest binding energy value, and its affinity is not in the catalytic cavity of MMP-9, but near Glu402 (Figure 4H). The catalytic pocket (Figure 4B–G) has hydrophobic interaction with different amino acids residues, particularly Pro430 (Fragment 1, 2, and 7), Asp410 and Asp434 (Fragment 4 and 5), Glu416 (Fragment 3), and Ser412 (Fragment 6), all this given by hydroxyl groups of each carotenoid chain.

#### 3.2.3. Molecular Docking and Molecular Dynamics Simulations of MMP-9 and Bacterioruberin

The complete molecule of Bacterioruberin was analyzed using a docking and molecular dynamics approach. Table 3 presents a summary of the results obtained during the molecular docking analysis. We tested three possible binding sites between Bacterioruberin and MMP-9, which are identified as Binding Site 1, Binding Site 2, and the ‘Allosteric Binding Site’.

Binding Site 1 and Binding Site 2 were identified as cavities using the Protein Plus Web Server (see Supplementary Material). The Allosteric binding site was previously described [29] during the development of an antiMMP-9 antibody. It should be noted that Binding Site 1 has a larger cavity than Binding Site 2 (see Supplementary Material). All three binding sites exhibited very similar coupling scores, which could suggest nonspecific binding by the ligand. Additionally, the primary residues with which bacterioruberin might interact are illustrated.

**Figure 5:**
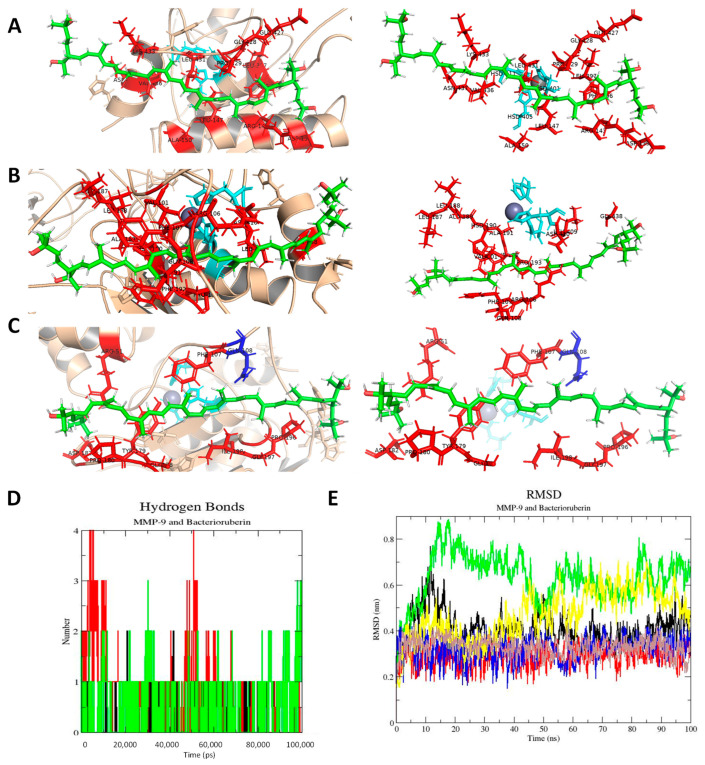
Molecular docking and molecular dynamics of bacterioruberin: (**A**) Binding site 1. (**B**) Binding site 2. (**C**) Allosteric Binding Site. Bacterioruberin colored in green, binding residues in red, active site in cyan, zinc in grey, Q108 in blue (a residue implicated in allosteric binding of an antibody reported by Appleby [29]). (**D**) H-bonds graph of the three binding sites analyzed for MMP-9 with bacterioruberin: Binding site 1 (black), Binding site 2 (red), and Allosteric Binding Site (green). (**E**) RMSD of MMP-9 and Bacterioruberin. MMP-9: Binding Site 1 (black), Binding Site 2 (green), and Allosteric Binding Site (yellow). Bacterioruberin: Binding Site 1 (red), Binding Site 2 (blue), and Allosteric Binding Site (brown).

Figure 5A–C depict possible interactions of bacterioruberin at the three mentioned binding sites. Binding site 2 exhibits a greater number of hydrogen bonds than Binding Site 1. The RMSD graph indicates the stability of the ligand throughout the simulation. The simulation also suggests that bacterioruberin could block both entrance pockets that were tested. Due to its size, it appears impossible for the molecule to enter the two analyzed pockets.

## 4. Discussion

The carotenoid extracts of strains *Natronococcus* sp. TC6 and *Halorubrum tebenquichense* SU10 showed the typical bacterioruberin “three-finger” spectra [30] found in other halophilic archaea from *Halobacteriaceae* and *Haloferacaceae* families [31,32,33]. Different bacterioruberin (BAR) isomers were found in the samples of carotenoid pigments; the presence of Astaxanthin and β-carotene was confirmed. Isomers like 9-*cis*-BAR or all-*trans*-BAR, *cis*-BAR, and 13-*cis*-BAR found in extracts have been described in other archaeal pigments like *Haloferax volcanii, Haloferax mediterranei*, and *Natrobacterium gregory* [12,34]. In other halophilic archaea, particularly *Halobacterium salinarum* and *Halococcus morrhuae*, the presence of all-*trans* and *cis*-BAR isomers had been detected [11,35]. The presence of Ɣ-carotene in *Halorubrum tebenquichense* SU10 (Figure 2A) is associated with the carotenogenesis of the haloarchaea, derived from lycopene and then converted to β-carotene, a key in the synthesis of other carotenoids like astaxanthin also present in this haloarchaea [9]. The different bacterioruberin (BAR) isomers present in the samples of carotenoid pigments are associated with the presence of lycopene, it is possible to find BAR isomers 5-*cis* ≥ all-*trans* > 9-*cis* > 13-*cis* > 15-*cis* > 7-*cis* > 11-*cis* [36].

Bacterioruberin (BAR) derivatives found in the pigment extracts like monoanhydrobacterioruberin (MABR), bisanhydrobacterioruberin (BABR), and 2-isopentenyl-3,4 dihydrorhodopin (DIR) are reported in strains like *Halorubrum sodomense* and *Haloarcula vallismortis* [37,38]. BAR and β-carotene found in *Halorubrum tebenquichense* have been reported in *Halorubrum* sp. TBZ16 [39]. A major abundance of bisanhydrobacterioruberin, 2-isopentenyl-3,4 dihydrorhodopin, and monoanhydrobacterioruberin has been reported in most haloarchaea, and in the case of *Halorubrum* sp. genera it is common to find bisanhydrobacterioruberin as the more abundant C50 carotenoid [13,40]. In the case of astaxanthin found in *Halorubrum tebenquichense* SU10, other xanthophylls were reported in *Halobacterium* and *Haloferax* species, principally canthaxanthin or haloxanthin [41,42].

It has been shown that carotenoid extracts from several haloarchaea have antioxidant activities and are dose- and profile-dependent, particularly of isomers of bacterioruberin and other carotenoids [8]. DPPH scavenging activity of the two strains studied (*Natronococcus sp*. TC6 and *Halorubrum tebenquichense* SU10) were higher (above 90%) compared with pigments extracted from *Aquisalibacillus elongatus* MB592, *Haloarcula japonica* HM1, and *Halobacterium salinarum* HM2 that were able to inhibit 20, 30, and 80% percent, respectively [16]. Other pigment extracts from strains like *Halorubrum tebenquichense, Haloarcula japonica* HM1, and *Halobacterium salinarum* HM2 have been reported as good FRAP inhibitors (above 70%) [16,43]. Contrary, for ABTS radical bleaching, *Natronococcus* sp. showed the best results when compared with *Halorubrum tebenquichense* SU10 and other strains of the same genus [16]. According to the antioxidant studies (Figure 3), the pool pigments of *Natronococcus* sp. TC6 and *Halorubrum tebenquichense* SU10 have a major antioxidant capacity of DPPH > ABTS > FRAP dominating the capacity of hydrogen and single electron transfer (radical neutralization behavior) as the typical “free radical scavenger” mechanism [44,45]. In addition, the antioxidant capacity was also influenced by strain type (Figure 3A–C). It had been shown that in combination, carotenoids can exert synergistic antioxidant activities, e.g., the mix of xanthophylls like astaxanthin and carotenes like lycopene (the two found in haloarchaea) act as molecular wiring, where the hydrophilic xanthophylls are the first step of the electron transfer and then the lipophilic carotenoids enhance the antioxidant capacity [31]. Additionally, the presence of xanthophylls can be associated with photoprotection, membrane stabilization, and protein-membrane function, this last item very important in haloarchaea [42]. This can be associated with the results of pool pigments in *Halorubrum tebenquichence* SU10 on FRAP that shows more antioxidant capacity than *Natronococcus* sp. TC6, related to the presence of astaxanthin in *Halorubrum* pigment extract and its participation in a synergy with the other pigments.

The MMP-9 is an enzyme of metalloproteinase group (MMP’s) particularly zinc (Zn^2+^) and calcium (Ca^2+^) dependent, present intracellularly and membrane-bound. Their importance is their participation in physiological processes including development, wound, healing, remodeling of tissues, angiogenesis, and particularly participation in the tumor invasion, metastasis, and angiogenesis acting in the degradation of gelatin and type IV, V, XI, and XII collagens. The overexpression of MMP-9 is associated with different cancers, such as lung, prostate, gastric, pancreatic, and colon and with other diseases, such as autoimmune and cardiovascular diseases [46,47]. The catalytic domain has 170 amino acids with a spherical structure of 40 Å in diameter, two zinc and calcium ions are present for catalytic activity and integrity, respectively [47].

The inhibitory effect of haloarchaeal pigment fragments over MMP-9 was suggested by docking assays. By this analysis, it is possible to identify from a pool of carotenoids which has a major capacity for MMP-9 inhibition. All fragments show H-bonds, given by hydroxyl groups, this behavior and inhibition type were similar for the fragments of bacterioruberin, monoanhydrobacterioruberin, and bisanhydrobacterioruberin, interacting with different amino acids in the catalytic pocket.

Docking studies point towards the importance of the archaeal carotenoids for MMP-9 inhibition because of their interaction in the catalytic pocket or around of active site. The inhibitory effect of archaeal carotenoids on MMP-9 has been studied in vitro using a carotenoid extract of *Natrialba* sp. M6, showing a repression of 50% in MMP-9 (0.5 µg/mL) [48]. However, in this study, is possible to show the specific inhibitory effect of each archaeal carotenoid fragment present in a pigment extract containing a pool of pigments. In this case, bacterioruberin, monoahydrobacterioruberin, and trisanhydrobacterioruberin from *Natronococcus* sp. TC6 and *Halorubrum tebenquichense* SU10 extracts showed the highest binding energies.

There are few reports about the molecular dynamics of bacterioruberin and other related carotenoids. The potential with molecular dynamics studies for other enzymes has been reported, like acetylcholinesterase (ACE) and butyrylcholinesterase using fragments of the carotenoids and showing binding energies in a range of ΔG (kcal/mol) −7.42 to −10.35 ΔG [16]. Molecular dynamics and docking studies of the complete bacterioruberin molecule were conducted, involving the selection of three binding sites. Two of these sites are located near the catalytic pocket, while the third is positioned near a binding site previously reported for the allosteric inhibition of MMP-9 by an antibody [29]. All three binding sites had very similar coupling scores, suggesting potential nonspecific binding by the ligand. Amino acids participating in these binding sites are predominantly hydrophobic, implying that the interaction between bacterioruberin and MMP-9 primarily occurs through hydrophobic interactions. This is consistent with the hydrophobic nature of bacterioruberin, and the presence of hydrophobic regions in the tested areas of MMP-9, as calculated by UCSF Chimera (see Supplementary Material).

Molecular dynamics results suggest that bacterioruberin blocks the entry to both Binding Site 1 and Binding Site 2. The selection of the Allosteric Binding Site was based on bacterioruberin large size. Previous crystallographic studies have confirmed MMP-9 interactions with large molecules, such as antiMMP-9 antibodies, making it plausible for bacterioruberin to interact in that area as well. As mentioned earlier, most of the residues involved in these interactions are primarily hydrophobic, indicating hydrophobic interactions as the primary mode, with some hydrogen bond formation (see Figure 5D). Additionally, the RMSD graph confirms the ligand stability throughout the 100 ns simulation.

The carotenoids profile of both strains was elucidated, and their antioxidant activity and MMP-9 inhibition in silico were evaluated, future research must be focused on elucidating the mechanism to enhance bacterioruberin and derivates production at a large scale and their incorporation in functional foods and ingredients.

## 5. Conclusions

*Halorubrum tebenquichense* SU10, isolated form Uyuni saltern in Bolivia and *Natronococcus* sp. isolated from El Golea, a salt lake in Argelia were characterized in their carotenoids pigments. Bacterioruberin was identified in both *Halorubrum tebenquichense* SU10 and *Natronococcus* sp. TC6 pigment extracts, also, bacterioruberin derivates were identified, 2-isopentenyl-3,4 dihydrorhodopin, and monoanhydrobacterioruberin. Other pigments and intermediaries were identified, astaxanthin, β-carotene, phytoene, and lycopersene. The pigment extracts showed considerable antioxidant activity on DPPH, ABTS, and FRAP. The inhibitory effect of the haloarchaeal pigments over MMP-9 was suggested by docking and molecular dynamics assays, monoahydrobacterioruberin and trisanhydrobacterioruberin display the best binding energy values. Molecular docking suggests that bacterioruberin has hydrophobic interactions with the tested MMP-9 sites, and the ligand remains stable during molecular dynamics simulations. Future research must be focused on large scale bacterioruberin production, also in the purification of pigments on the pool and their study on health benefits and also to verify their effect on MMP-9 inhibition on in vitro assays.

## Figures and Tables

**Table 1:** Identification of carotenoids produced by *Natronococcus* sp. TC6 and *Halorubrum tebenquichense* SU10 using UPLC-ESI-MS/MS technique.

Carotenoid Identified	Molecular Formula	Theoretical Mass (*m*/*z*)	Measured Mass (*m*/*z*)	Strain Pool
Bacterioruberin (BAR)	C_50_H_76_O_4_	740.57	740.5	Both strains
Monoanhydrobacterioruberin (MABR)	C_50_H_74_O_3_	722.56	722.47	*Nc*
Bisahnydrobacterioruberin (BHR)	C_50_H_72_O_2_	704.55	704.53	Both strains
Trisanhydrobacterioruberin	C_50_H_70_O	687.06	687.4	Both strains
2-isopentenyl-3,4 dihydrorhodopin (DIR)	C_45_H_64_O	620.96	620.3	*Hr*
Astaxanthin	C_40_H_52_O_4_	596.83	597.39	*Hr*
β-carotene	C_40_H_56_	536.87	537.2	Both strains
Phytoene	C_40_H_64_	544.94	543.50	*Nc*
Lycopersene	C_40_H_66_	547.0	545.96	*Nc*

*Nc*: *Natronococcus* sp. TC6, *Hr*: *Halorubrum tebenquichense* SU10.

**Table 2:** Binding energies and H-bonds obtained from molecular dockings of specific fragments of haloarchaea carotenoids (Figure 1) of *Natronococcus* sp. TC6 and *Halorubrum tebenquichense* SU10 over matrix metallopeptidase 9 (MMP-9).

Compound (Ligand)	Binding Energies (kcal/mol) Matrix Metallopeptidase 9 (MMP-9)	H-Bonds
Fragment 1, Bacterioruberin	−8.3	1
Fragment 2, Monoanhydrobacterioruberin	−8.2	1
Fragment 3, Bisanhydrobacterioruberin	−7.4	1
Fragment 4, 9-*cis*-26-*cis* bacterioruberin	−6.7	1
Fragment 5, 9-*cis*-bacterioruberin	−6.6	3
Fragment 6, 13-*cis*-bacterioruberin	−6.8	1
Fragment 7, Trisanhydrobacterioruberin	−8.3	1

**Table 3:** Binding energies and residues from molecular docking of bacterioruberin over matrix metallopeptidase 9 (MMP-9).

Binding Site	Binding Energies (kcal/mol)	Binding Residues
Binding Site 1	−5.3	D139, R143, L147, A150, F396, L397, P429, G428, E427, N437, K433, L431, V436
Binding Site 2	−5.1	V101, R106, F107, Q108, L187, L188, A189, H190, F192, G190, L409, D410, G438, P193
Allosteric Binding Site	−5.9	R51, F107, Q108, G178, Y179, P180, D182, P196, G197, I198

## Data Availability

Data supporting reported results can be found here: Haloarchaea pigments.

## Supplementary Materials

The following supporting information can be downloaded at: https://www.mdpi.com/article/10.3390/microorganisms11092344/s1.
