# Supplementary material for: Identification, Antioxidant Capacity, and Matrix Metallopeptidase 9 (MMP-9) In Silico Inhibition of Haloarchaeal Carotenoids from Natronococcus sp. and Halorubrum tebenquichense
Source: Microorganisms. 2023 Sep 19;11(9):2344. doi: 10.3390/microorganisms11092344 (PMC10537243; doi:10.3390/microorganisms11092344)
Supplement: Supplementary file 1 [file microorganisms-11-02344-s001.zip › microorganisms-2491365-supplementary.pdf]

SUPPLEMENTARY MATERIAL

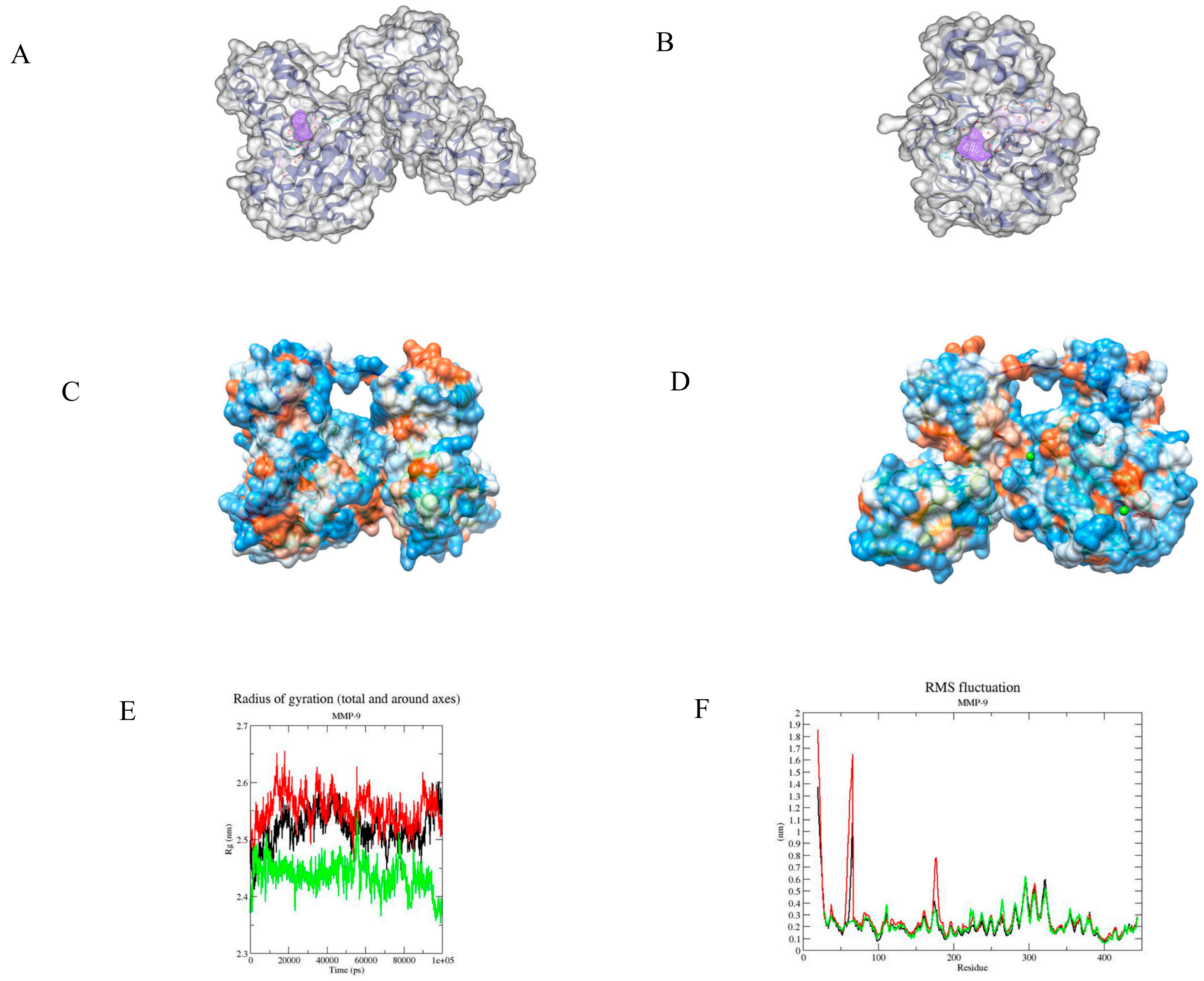

Figure S1.- Protein pockets calculated with Protein Plus server: (A) Binding site 1 and (B) Binding site 2.

Protein hydrophobic surface calculated with UCSF Chimera: (C) Back, Allosteric binding site region and (D) front, showing binding site 1 region. (blue=hydrophilic and orange=hydrophobic).

(E) Radius of gyration from MMP-9 calculated by molecular dynamic simulation: Binding site 1 (black), Binding site two (red) and allosteric binding site (green).

(F) RMSF of MMP-9 calculated by molecular dynamic simulation: Binding site 1 (black), Binding site two (red) and allosteric binding site (green).

Table S1.- Pocket properties calculated by Protein Plus server/

POCKET PROPERTIES	BINDING SITE 1	BINDING SITE 2
ACCEPTOR	11	7
DEPTH (Å)	12.92	6.30
DONORS	5	1
HYDROPHOBICITY	0.64	0.70
SURFACE (Å^2)	324.85	203.11
SURFACE VOLUME RATIO	1.59	2.31
VOLUME (Å^3)	204.80	88.06
